# The evaluation of the Myxovirus Resistance 1 protein in serum and saliva to monitor disease activation in primary Sjögren's syndrome

**DOI:** 10.6061/clinics/2019/e631

**Published:** 2019-06-11

**Authors:** Yasemin Gul Aydemir, Ahmet Kocakusak

**Affiliations:** I Clinic of Internal Medicine, Malkara State Hospital, Tekirdag, Turkey; II General Surgery Clinic, Health Ministry Haseki Teaching and Research State Hospital, University of Health Sciences, Istanbul, Turkey

**Keywords:** ESSDAI, Hydroxychloroquine, Myxovirus Resistance 1, Sjögren's syndrome

## Abstract

**OBJECTIVE:**

Primary Sjögren's syndrome (pSjS) is a chronic autoimmune disease that causes dry eye and mouth. No laboratory parameters to monitor the activation of this disease have been identified. Therefore, any possible relationships between salivary and blood myxovirus resistance 1 (MX1) and pSjS must be prospectively studied.

**METHODS:**

Thirty female patients with pSjS, 30 women with rheumatoid arthritis (RA) without secondary Sjögren's syndrome (SjS) and 28 healthy control women were enrolled in this investigation. Analyses of MX1 by the enzyme-linked immunosorbent assay (ELISA) method, SS-A (Ro) and SS-B (La) tests by the strip immunoblot method, anti-nuclear antibody (ANA) tests by immunofluorescence and the measurement of serum rheumatoid factor (RF), C3, C4, immunoglobulin A (IgA), immunoglobulin M (IgM), and immunoglobulin G (IgG) were performed.

**RESULTS:**

The serum level of MX1 in patients without Raynaud phenomenon was higher than in those with Raynaud phenomenon (*p*:0.029, *p*<0.05, statistically significant). There was a statistically significant positive association between hemoglobin levels and MX1 serum levels. No statistically significant association was found among the other parameters. Low MX1 levels were shown to be associated with both a low disease activity score based on the European League Against Rheumatism (EULAR) Sjögren's Syndrome Disease Activity Index (ESSDAI) and hydroxychloroquine use in all patients.

**CONCLUSION:**

MX1 levels have a considerable impact on the assessment of the disease activity in SjS. We believe that more-comprehensive studies should be performed on patients with pSjS who do not use hydroxychloroquine to prove this relationship and that MX1 levels should be used as a routine marker for the assessment of pSjS disease activity. Further studies are needed to create awareness of the role that MX1 has in the diagnosis of pSjS, which may help to uncover novel pathways for new therapeutic modalities.

## INTRODUCTION

Sjögren's syndrome (SjS) is a slowly progressive chronic autoimmune disease that causes symptoms including dry mouth (xerostomia) and dry eye (keratoconjunctivitis sicca), which is mainly attributed to lymphocytic invasion of the exocrine glands 1. To clinically monitor the disease, the European League Against Rheumatism (EULAR) Sjögren's Syndrome Disease Activity Index (ESSDAI) has been used to measure disease activation. However, no clearly described laboratory parameters to monitor disease activation have been validated 2. The plasma of patients with primary SjS (pSjS) contains high levels of interferon (IFN)-α. Moreover, IFN-α increases the mRNA levels in circulating blood cells. IFN-α-containing epithelial cells and lymphocytes have been found in the salivary glands. The IFN source is probably the plasmacytoid dendritic cells that collect in the salivary glands. In monocytic cell lines, increased levels of the genes that regulate the expression of type 1 IFN have been observed; additionally, the high bioactivity of type 1 IFN has been observed in the serum of patients with pSjS. Myxovirus Resistance 1 (MX1, also known as MxA) is the main mediator of IFN-based antiviral reactions, and MX1 is strongly affected by type 1 IFN. Studies have shown that MX1 gene expression in systemic sclerosis (SS) is a kind of type 1 IFN bioactivity that is associated with disease activity. These findings have also shed light on the relationship between high disease SS activity scores in the ESSDAI and type 1 IFN, and MX1 is a potential biomarker for SS. In the literature, a cohort study with a total of 35 patients with pSjS, of whom 21 were IFN-positive (having an IFN score of 10 and higher) and 14 were interferon-negative (having an IFN score of less than 10), showed a significant positive association between the IFN scores, which were used to measure the bioactivity of type 1 IFN, the ESSDAI disease activity scores and the serum levels of MX1 3.

In light of this information, we aimed to assess any relationship between serum and saliva MX1 protein levels and disease activity in pSjS patients. The primary endpoint was defined as the assessment of the relationship between MX1 protein levels and ESSDAI scores. In this context, we aimed to evaluate whether the serum or saliva MX1 protein was ideal for use in clinical practice as a laboratory parameter to monitor disease activity and the response to drug therapy. As secondary endpoints, we aimed to assess the relationship between MX1 and the serum SS-A (Ro) antibody, serum SS-B (La) antibody, serum anti-nuclear antibody (ANA), serum rheumatoid factor (RF) antibody, immunoglobulin G (IgG), immunoglobulin M (IgM), and immunoglobulin A (IgA), which were previously shown to be important for the detection and monitoring of pSjS and for describing the relationship between MX1 and other clinical findings. Until now, there has been no laboratory marker available for assessing disease activation in pSjS 2,4.

## PATIENTS AND METHODS

All procedures were approved by and performed in accordance with the ethical standards of the responsible committee on human experimentation (institutional) and were in accordance with the Declaration of Helsinki. The ethics committee approval for the study was given by Yeditepe University Institutional Ethics Committee in 2015, and the doctoral thesis of Yasemin Gul AYDEMIR was conducted at the same university. Moreover, informed consent forms were signed by all participants.

From February 2015 to February 2016 (within a 1-year follow-up period), 30 female pSjS patients, who were diagnosed based on the 1996 European classification norms, were followed up at the rheumatology clinics of 2 university hospitals (Yeditepe University Hospital and Istanbul Bilim University Hospital) and received hydroxychloroquine treatment, were enrolled. Additionally, 30 women with rheumatoid arthritis (RA) without evidence of secondary SjS and 28 healthy control women were enrolled. Hence, 88 women (age range: 21-64 years) were evaluated herein in a prospective and descriptive investigation. Based on their previous medical histories, patients were excluded for the following reasons: lymphoma, graft-versus-host disease, radiotherapy to the head and neck, AIDS, sarcoidosis, hepatitis C infection, and the use of anticholinergic drugs (neuroleptics, antidepressants, antihypertensives, and parasympatholytics). The demographic characteristics, past medical history and family medical histories of the patients (age, gender, profession, age of onset of the disease, family history, systemic diseases, medications, radiation history, smoking habits, and alcohol consumption) were recorded in detail, and a detailed physical examination of the patients was performed at all centers. The assessment of disease activity was performed with the ESSDAI in patients with pSjS. Sociodemographic data, symptoms, the duration of symptoms, age of diagnosis, laboratory findings, physical examinations, treatment and disease activity assessment information were obtained after patients completed the general consent form. When the local ethics committee had approved the study, serum and saliva samples were taken from all the volunteers. The analysis of MX1 by the enzyme-linked immunosorbent assay (ELISA) method, SS-A (Ro), SS-B (Lo) tests by the strip immunoblot method, an anti-nuclear antibody (ANA) test by immunofluorescence and the measurement of the concentrations of serum RF, C3, C4, IgA, IgM and IgG by a nephelometric method from the serum and saliva samples were performed by the Tissue Typing Laboratory of Yeditepe University Hospital.

After collecting blood for hemoglobin testing, two extra tubes with latex were used to collect 5 mL of blood from each volunteer. Blood samples were centrifuged at 3000 rpm for 10 minutes in the Yeditepe University Hospital Stem Cell Laboratories after being incubated for half an hour at room temperature to obtain the patients' serum. Saliva samples (2 ml) were taken between 9-11 AM, at least 2 hours after stopping food and beverage intake. After rinsing their mouths with water, volunteers were asked to collect the spit over 5 to 15 minutes. The 2-ml saliva samples were aliquoted into five separate, capped 1.5-ml Eppendorf tubes. The aliquots of the serum and saliva samples were stored at the Yeditepe University Hospital Stem Cell Laboratory at -80°C until further analysis.

ELISA tests: The MX1 protein from blood and saliva samples was tested in accordance with the kit recommendations by the ELISA (Elabscience® ELISA Kit) method. The ELISA method has been used in our laboratory for a long time. Briefly, test sera was added to 96-well ELISA plates that were coated with antigen or antibody and was incubated for a certain period of time. Then, the plates were washed twice, a second incubation with secondary antibodies was performed, and colorimetric reagent was added. After a short incubation period, the reaction was stopped with the addition of a reagent, and spectrophotometric measurement (Epoch ELISA reader) was performed within no more than 10 minutes. The quantitation was automatically performed by the plate reader according to the standard reagent on the plate. One of the most important criteria for the success of ELISA tests is accurate pipetting. In our laboratory, the pipettes are regularly calibrated. The researchers also received detailed training in this regard. The detection range of the ELISA Kit for MX1 was 1.56-100 ng/mL, and the test method was a double-antibody sandwich. In regard to sensitivity, the minimum detectable dose was less than 0.65 ng/mL.

Immunoblot method tests: SS-A (Ro) and SS-B (La) tests were performed using the strip immunoblot method.

Immunofluorescence tests: ANA immunofluorescence tests were performed by microscopic examination.

Nephelometric measurements: Serum RF, C3, C4, IgA, IgM and IgG concentrations were measured by a nephelometric method.

Statistics: When statistically evaluating the findings obtained in this study, the SPSS 22 (IBM, Turkey) program was used. The reliability of the distribution of the parameters was evaluated by the Shapiro-Wilks test. One-way ANOVA was used for descriptive statistics, such as the standard deviation, mean, and frequency, and the two-way intergroup comparisons of quantitative data were evaluated. When two groups with a normal distribution were compared, a Student's t test was used. However, for abnormally distributed data, the Mann-Whitney U test was used to compare two groups. Pearson correlation analysis was used to evaluate the relationships between the parameters. A *p*<0.05 was considered significant.

## RESULTS

The patients' mean age was 48.00±9.78 years. There was not a significant difference in the mean age among the groups (*p*>0.05). The age of the patients in the pSjS group ranged from 30 to 64 years, and the mean age was 51.07±10.17 years. The age of the patients in the RA group ranged from 21 to 62 years, and the mean age was 47.27±11:55 years. The age of the patients in the control group ranged from 33 to 54 years, and the mean age was 45.39±5.96 years.

When evaluating patients with and without pSjS, the durations of the diseases were 7.57±6.79 and 9.64±6.89 years, respectively. Ocular and oral symptoms were present in all pSjS patients. The ocular test results showed Schirmer <5 in 24 (80%) pSjS patients and Schirmer >5 in the remaining 6 (20%) pSjS patients. The following results were noted in the pSjS patients: positive histopathology in 6 (20%), positive salivary gland test results (sialometry and scintigraphy) in 30 (100%), pulmonary involvement in 2 (7%), lymphadenopathy in 9 (30%), parotid gland involvement in 2 (7%), arthralgia or arthritis in 23 (77%), skin involvement in 8 (27%), cryoglobulinemia in 4 (13%), Raynaud phenomenon in 14 (47%), the presence of the SS-A antibody in 20 (67%), the presence of the SS-B antibody in 20 (67%), ANA positivity in 28 (93%), and RF in 4 (13%). While all patients (n: 30) with pSjS were given hydroxychloroquine, 2 (7%) were additionally treated with pilocarpine, and 2 (7%) were also treated with corticosteroids.

No significant difference was detected between the pSjS and RA groups with regard to the mean serum or saliva MX1 values (*p*>0.05). In regard to the disease duration, there was no significant difference between the pSjS and RA patient groups (*p*>0.05). When serum and saliva MX1 values were compared with sialometry results, ESSDAI scores, and Schirmer values, no correlations were detected. In the pSjS group, a positive association was detected between the serum values of MX1 and hemoglobin levels, and this correlation was statistically significant. The serum MX1 values in patients without Raynaud phenomenon were higher than those in patients with Raynaud phenomenon, and this association was statistically significant. The differences between the other parameters were not statistically significant.

There was not a statistically significant difference between the pSjS and RA groups with regard to the disease duration (*p*>0.05). The disease duration of the pSjS group ranged from 0.1 to 24 years, with a mean of 7.57±6.79 years. In the RA group, the disease duration range was 0.1 to 20 years, with an average of 4.89±5.39 years. The symptom duration of the pSjS group ranged from 0.1 to 24 years, with a mean of 9.64±6.89 years.

There were not statistically significant differences between the groups in terms of the mean serum and salivary MX1 levels (*p*>0.05) (Table 1).

There was no statistically significant relationship between the serum levels of MX1 and the sialometry results; ESSDAI scores; and neutrophil, hemoglobin and sedimentation levels in the pSjS group (*p*>0.05) (Table 2).

There was no statistically significant difference (*p*>0.05) among the MX1 serum values according to the ESSDAI scores and Schirmer test results in the pSjS group. There was not a statistically significant difference (*p*>0.05) between the serum levels of MX1 according to the presence of lymphadenopathy, parotid involvement, arthralgia/arthritis, skin involvement, pulmonary involvement, cryoglobulinemia, hypocomplementemia and neutropenia. However, the mean serum level of MX1 in patients with no Raynaud phenomenon was significantly higher than that in patients with Raynaud phenomenon (*p*:0.029, *p*<0.05) (Table 3).

There were no statistically significant correlations between the MXI salivary values and the sialometry results and ESSDAI scores in the pSjS group (*p*>0.05).

There were no statistically significant differences between the MX1 salivary values according to the ESSDAI scores and Schirmer test results (*p*>0.05) in the pSjS group.

There were no statistically significant differences (*p*>0.05) between the serum levels of MX1 according to the presence of anti-SS-A, anti-SS-B, RF and ANA (Table 4).

There were no statistically significant associations between the serum levels of MX1 and the C3, C4, IgA, IgG, and IgM levels (*p*>0.05). However, there was a statistically significant positive association between hemoglobin levels and serum MX1 levels (27.3%) (r:0.273, *p*:0.035, *p*<0.05) (Table 5).

## DISCUSSION

SjS causes lymphocytes to invade the salivary and lacrimal glands. Therefore, SjS is known as an autoimmune systemic disease characterized by dry mouth and eye. If the patient does not have another autoimmune disease, SjS is defined as pSjS. SjS is defined as secondary SjS if the patient has another autoimmune disease, including RA or systemic lupus erythematosus (SLE). Although this disease can be diagnosed in patients of all ages, it is usually diagnosed between the 4^th^ and 6^th^ decades. SjS is more common in females than in males, and is diagnosed with a 9:1 female:male ratio 5.

The etiopathogenesis of pSjS is multifactorial. Genetic susceptibility and hormonal and environmental contributors have been implicated 6. The ESSDAI has been used to assess the disease activity. The ESSDAI evaluates the organs that are affected that contribute to disease activity in approximately 30% of patients, including pulmonary findings, vasculitis, synovitis, peripheral and central nervous systems findings, and renal and hematological system symptoms. In addition to assessing disease activation, treatment success is also monitored by the ESSDAI. Until now, there has unfortunately been no laboratory available marker for measuring disease activation 7.

MX1 is the main mediator of the interferon-based reaction against viruses and is strongly regulated by type 1 IFN. In previous studies, MX1 gene expression in SS was shown to be a type 1 IFN bioactivity and was associated with disease activity. In a study of 50 scleroderma patients, an increase in MX1 expression was associated with severe disease findings, such as decreased vital capacity and digital ulcers. MX1 has been identified as the most practical biological marker to evaluate IFN type I bioactivity in pSjS 8.

These findings suggested that high ESSDAI disease activity scores in SjS may be related to a potential biomarker of type 1 IFN, MX1. In a single study in the literature, researchers evaluated 21 IFN-positive (IFN score of 10 and more) and 14 IFN-negative (IFN score below 10) patients with pSjS. These researchers compared the 35 patients who had been diagnosed according to 2002 American-European criteria and treated with high-dose prednisolone (more than a 10 mg daily dose) without immunosuppressive drug and biologic drug use with 27 healthy individuals without autoimmune disease and with no corticosteroid use. These researchers found a significant positive relationship between the IFN scores and ESSDAI disease activity scores that were used to measure the total type 1 IFN bioactivity and serum MX1 levels. Maria et al. 3 found high MX1 levels in active pSjS patients and found an association between high MX1 levels with higher ESSDAI scores, RF, SS-A or SS-B antibodies, and immunoglobulin but with lower hemoglobin and neutrophil values. At the end of this study, these researchers suggested that MX1 might be a biomarker for disease activation. Interestingly, in the same study, the MX1 levels were lower in the group using hydroxychloroquine than in the other patient groups. This could explain our nonsignificant results.

In the present study, we evaluated the effectiveness of the MX1 protein in both the saliva and serum samples of pSjS patients for the detection of disease activation. Contrary to the study described above, we did not find any relationship between serum and saliva MX1 levels and disease activity. All of our patients with pSjS were using hydroxychloroquine. However, in the study by Maria NI et al. 3, serum MX1 values were also found to decrease in patients treated with hydroxychloroquine. These authors suggested that MX1 might be useful for predicting the therapeutic response to hydroxychloroquine. In another study, hydroxychloroquine was shown to affect systemic IFN formation 9. In our patient group, the use of hydroxychloroquine and the low ESSDAI disease activity scores in all patients may explain the low MX1 levels that we detected. Malignancy has also been widely discussed in the literature because the chronic use of hydroxychloroquine, especially for gastrointestinal manifestations, hinders autophagy and systemic inflammation, thus probably having some antineoplastic effects. However, these theories are still debated 10,11.

Recent studies have shown that the activated type 1 IFN pathway plays a crucial role in autoimmune diseases 4. In a study that used an ELISA and real-time PCR to measure type I IFN expression at the transcriptional and protein levels in labial salivary gland biopsies, plasma, and peripheral blood cells and performed immunohistochemistry in pSjS samples, it was found that the IFN-α level was high in the patients' labial salivary glands, as well as in their plasma and peripheral blood cells 5. SLE, SjS, dermatomyositis, and psoriasis are the main diseases in which the IFN pathway has been shown to important in genetic studies. In particular, the study of SLE provide additional beneficial information to aid in the understanding of the pathogenesis of pSjS 6,7. For example, the transactivation of type I IFN and IFN-inducible genes causes the polymorphism of interferon regulatory factor (IRF5), which is also known to be related to SLE. In addition, a relationship between the CGGGG insertion or deletion polymorphism of the IRF5 promoter and pSjS was identified. Increased mRNA levels of IRF5 in circulating blood mononuclear cells and in the epithelial cells of the salivary glands in patients who had four repeats of the CGGGG IRF5 allele were positively correlated with the levels of mRNA for IFN-inducible genes and MX1 8,9.

The limitation of this study, which also negatively influenced our results, was that the patient group was being treated with hydroxychloroquine before, during and after our measurements, since the ethics committee did not permit us to discontinue the drug during the study. However, a reliable marker should not be affected by treatment methods. Moreover, the evaluation of the patients admitted to only two university hospitals resulted in a randomization bias, as is the case in most similar studies. However, MX1 was detected at low levels despite both the low disease activity score and hydroxychloroquine use 10. Since a minority of patients had high ESSDAI scores, the analysis of the relationship of ESSDAI scores with MX1 may have been impaired. The clinical and laboratory parameters as well as the ESSDAI levels were assessed in some of the pSjS patients. However, it is unlikely that the increased ESSDAI scores were an effect of reduced hydroxychloroquine use. Few studies have been conducted on this topic. When compared with those of other studies, our results may have been affected by the use of hydroxychloroquine 11.

In conclusion, in the present study, we evaluated the relationship between serum and saliva MX1 levels and disease activity scores (ESSDAI) in patients with pSjS. To date, there has been no laboratory marker available for measuring disease activation. In the SjS group, there was a statistically significant positive correlation between the serum levels of MX1 and hemoglobin levels (27.3%). The mean serum level of MX1 in patients who were negative for Raynaud phenomenon was significantly higher than that in patients who were positive for Raynaud phenomenon. This difference had a very borderline level of statistical significance, and there was a very small difference in the MX1 levels. No statistically significant association was found in the other clinical and laboratory parameters. We believe that the low MX1 levels in our series suggest that this marker can only be used in a select group of patients, such as for monitoring the effect of hydroxychloroquine in patients with high MX1 levels before treatment begins. MX1 levels play a considerable role in impacting the disease activity assessment of SS. We believe that more comprehensive studies should be carried out in patients with pSjS who do not use hydroxychloroquine to prove these relationships for MX1 levels to be used as a routine marker for the assessment of pSjS disease activity. Further studies are necessary on both female and male patients with pSjS who do not use hydroxychloroquine to better understand the relationship between the diagnosis of pSjS and MX1, which may reveal novel potential pathways for new therapeutic modalities.

## AUTHOR CONTRIBUTIONS

Aydemir YG planned the experiments and methodology, in addition to researching techniques. Kocakusak A generated data collection forms, analyzed the data, and wrote and edited the manuscript. Additionally, Kocakusak A evaluated the quality of the study, made overall assessments in regard to analyzing and presenting its contents and conclusions, and accepting and rejecting items or materials and patients. Finally, Kocakusak A reviewed, drafted and submitted the study. Both authors performed and approved critical revisions of the manuscript.

## Figures and Tables

**Table 1:** The evaluation of the serum and saliva levels of MX1 in the different groups (One-way ANOVA test).

	pSjS (n=30)	RA (n=30)	Control (n=28)	*p*
	Mean±St. Dev.	Mean±St. Dev.	Mean±St. Dev.	
MX1 serum	0.09±0.02	0.09±0.03	0.09±0.02	0.646
MX1 saliva	0.09±0.03	0.09±0.02	0.09±0.02	0.913

**Table 2:** The correlation analysis of serum MX1 and clinical parameters in the pSjS group (Pearson correlation analysis).

pSjS	MX1 Serum	
	r	p
Sialometry	0.056	0.768
ESSDAI score	0.113	0.552
Neutrophils	0.128	0.502
Hemoglobin	0.173	0.359
Sedimentation	0.221	0.241

**Table 3:** The MX1 serum evaluation in the pSjS group according to the clinical parameters.

pSjS		MX1 Serum	*p*
		Mean±St. Dev.	
ESSDAI score	<7 (n=28)	0.09±0.02 (0.08)	^1^0.560
	>7 (n=2)	0.09±0.01 (0.09)	
Schirmer test	<5 (n=24)	0.09±0.02 (0.08)	^1^0.312
	>5 (n=6)	0.08±0.01 (0.08)	
Raynaud phenomenon	Positive (n=14)	0.08±0.01	^2^0.029*
	Negative (n=16)	0.09±0.03	
Lymphadenopathy	Positive (n=9)	0.08±0.02 (0.08)	^1^0.377
	Negative (n=21)	0.09±0.02 (0.08)	
Parotid involvement	Positive (n=2)	0.11±0.03 (0.11)	^1^0.074
	Negative (n=28)	0.08±0.02 (0.08)	
Arthralgia/Arthritis	Positive (n=23)	0.08±0.02 (0.08)	^1^0.641
	Negative (n=7)	0.09±0.02 (0.09)	
Skin involvement	Positive (n=8)	0.08±0.02 (0.08)	^1^0.280
	Negative (n=22)	0.09±0.02 (0.02)	
Pulmonary involvement	Positive (n=2)	0.08±0.01 (0.08)	^1^0.677
	Negative (n=28)	0.09±0.02 (0.08)	
Cryoglobulinemia	Positive (n=4)	0.09±0.01 (0.08)	^1^0.760
	Negative (n=26)	0.09±0.02 (0.08)	
Complement allowance	Positive (n=4)	0.08±0.01 (0.08)	^1^0.502
	Negative (n=26)	0.09±0.02 (0.08)	
Neutropenia	Positive (n=13)	0.08±0.02	^2^0.162
	Negative (n=17)	0.09±0.03	

(^1^Mann-Whitney U test, ^2^Student’s t test, *p<0.05)

**Table 4:** The evaluation of serum MX1 compared to autoantibody levels (Student’s t test).

		MX1 Serum	*p*
		Mean±St. Dev.	
Anti-RO (SS-A antibody)	Positive (n=23)	0.08±0.02	0.353
	Negative (n=37)	0.09±0.03	
Anti-LA (SS-B antibody)	Positive (n=18)	0.09±0.02	0.787
	Negative (n=42)	0.09±0.03	
RF	Positive (n=27)	0.09±0.03	0.098
	Negative (n=33)	0.08±0.02	
ANA	Positive (n=49)	0.08±0.02	0.072
	Negative (n=11)	0.10±0.04	

**Table 5:** The correlation analysis of serum MX1 levels and laboratory parameters (Pearson correlation analysis, *p<0.05).

	MX1 Serum	
	r	*p*
Hemoglobin	0.273	0.035*
C3	0.013	0.944
C4	0.229	0.223
IgA	0.112	0.555
IgG	0.007	0.969
IgM	0.147	0.438
